# Early life factors associated with the experiences of pain in later life: evidence from a population based study in India

**DOI:** 10.1186/s12889-023-15805-6

**Published:** 2023-05-26

**Authors:** Waquar Ahmed, Manacy Pai, T. Muhammad, Chanda Maurya, Parimala Mohanty, Nargis Begum Javed

**Affiliations:** 1 School of Health Systems Studies, Tata Institute of Social Sciences, Mumbai, India; 2 Department of Sociology and Criminology, Kent State University, Kent, OH 44242 USA; 3 Department of Family & Generations, International Institute for Population Sciences, Mumbai, India; 4 Department of Survey Research and Data Analytics, International Institute for Population Sciences, Mumbai, India; 5 Institute of Medical Sciences … Sum Hospital, Siksha “O” Anusandhan Deemed to be University, Bhubaneswar, Odisha India; 6 Department of Public health, College of Health sciences, Saudi Electronic University, Dammam, Saudi Arabia

**Keywords:** Early-life factors, Pain experience, Older adults, India

## Abstract

**Background:**

The influence of early life factors is becoming increasingly apparent as studies investigate how experiences, resources, and constraints in childhood affect health and well-being later in life. The present study contributes to this literature by examining the association between several early life factors and self-reported pain among older adults in India.

**Methods:**

Data come from the 2017-18 wave 1 of the Longitudinal Ageing Study of India (LASI). The sample size includes 28,050 older adults aged 60 and above (13,509 men and 14,541 women). Pain is a self-reported, dichotomous measure where participants responded to whether they were often troubled with pain and whether this experience interfered with their ability to carry out daily household chores. Early life factors, which are retrospective accounts of experiences, included the respondent’s position in birth order, their health status, school absenteeism, being bedridden, family socioeconomic status (SES), and their parent’s experience with chronic disease. Logistic regression analysis is employed to examine the unadjusted and adjusted average marginal effects (AME) of selected domains of early life factors associated with the probability of experiencing pain.

**Results:**

22.8% of men and 32.3% of women reported pain that interfered with daily activities. Pain was higher among men (AME: 0.01, confidence interval (CI): 0.01–0.03) and women (AME: 0.02, CI: 0.01–0.04) with third or fourth birth order compared to counterparts with first birth order. Both men (AME: -0.02, CI: -0.04–0.01) and women (AME: -0.07, CI: -0.09 - -0.04) having a fair childhood health status reported a lower probability of pain. The probability of pain was higher among both men (AME: 0.03, CI: 0.01–0.07) and women (AME: 0.07, CI: 0.03–0.13) who were bedridden due to sickness in their childhood. Similarly, the pain likelihood was higher among men who missed school for more than a month due to health problems (AME: 0.04, CI: -0.01-0.09). Men and women with poor financial condition in their childhood reported (AME: 0.04, CI: 0.01–0.07) a higher probability of experiencing pain relative to their peers who reported a more financially advantaged early life.

**Conclusions:**

Findings of the present study add to the empirical literature on the association between early life factors and later life health and well-being. They also are pertinent to health care providers and practitioners working in pain management, as this knowledge better positions them to identify older adults most susceptible to pain. Moreover, findings of our study underscore that the interventions to ensure health and well-being in later life must start far earlier in the life course.

## Background

Chronic pain is among the most prevalent and serious health conditions worldwide and a primary cause of years lived in disability [1]. It is particularly common and challenging in older adults, aged 65 and above [2], as it is associated with increased functional limitations, dependence, and social isolation, a decreased quality of life, and poor mental health [2–6]. Pain that interferes with daily life or work activities can also be financially burdensome for families, health-care systems, and society at large [2]. Worse, chronic pain significantly increases the risk of cognitive impairment [7] and premature death [8].

The etiology of pain reflects a variety of intricate and evolving interactions between biological, psychological, and social stressors operating through a range of mechanisms over the course of one’s life [9, 10]. As such, an increasing number of studies are focusing on factors outside of the contemporaneous life stage to understand the long-term correlates of later life pain. Empirical evidence continues to point out relevance of early life circumstances to vulnerability to pain in later life [11]. Based on the psychoneuroimmunological approach, early life adversity may produce altered or blunted stress axis reactivity, physiological sensitization to stress, and dysregulation of the immune system [12–16]. Dysregulation of the stress and immune systems, in turn, is linked with elevated and prolonged inflammation [15, 17, 18], which may result in pain sensation [19].

Socioeconomic disadvantage in childhood, for example, may physiologically manifest as exaggerated or diminished stress reactivity in children [16], which can build over the course of a person’s life, increasing their susceptibility to chronic illnesses [20], including body pain. Those who experience chronic stress early in life owing to financial hardship are more susceptible to a series of stressful events (such as family conflict, food insecurity, substance use, and neighborhood disorder), which also increases the likelihood of bad health in later life [20]. Early life socioeconomic disadvantage may persist into early adulthood, which may negatively affect health in later life [21]. Further, relative to their well-to-do peers, children of lower socioeconomic status (SES) perform less well academically, which may preclude them from amassing the necessary human and social resources and the cultural health capital necessary for maintaining good health at older ages [21].

Similarly, the experience of health problems in early life is linked with chronic pain in older adults [22]. For instance, in a British cohort study that was followed up since birth, the occurrence of serious illness before the age of 25 was found to be associated with an increased risk of chronic widespread pain at age 68 [22]. Having health problems in early life, being bedridden, and missing school can be accompanied by poor academic performance, which is directly and indirectly linked to subsequent health troubles [21, 23]. Aside from school absenteeism and poor academic performance, the severity of illness and complications associated with treatment, including side-effects of medication, are known to produce cognitive delays in children and youth [24, 25], which may render them susceptible to all sorts of health troubles, including chronic disease and pain in later life. Early life exposure to financial and health stressors may also indirectly shape later life health through their influence on educational attainment, employment stability, economic security, and also the ability to develop and maintain social relationships [26, 27], the latter being critical for later life cognitive functioning [28].

Birth order is another factor that has received less attention in the literature on early life conditions and subsequent health. Because of biological differences or maternal behavior, siblings may have different prenatal environments despite having the same gene pool [29]. Birth order may determine the extent of direct and indirect health, emotional, and human capital investments parents make in each child [29–33], which may exert an impact on the health of children well into adulthood and later life. Also, there is some data that suggest that later-borns partake in unhealthy activities and invest less in human and health capital [34], both of which are required to sustain good health across the life course.

In addition to one’s own early life health problems, parental health also is an important predictor of adult health [35, 36]. For instance, a parent’s capacity to properly care for and supervise children may be hampered by a physical or mental health condition (e.g., depression, cancer, or cardiovascular disease) [37]. Maternal depression, for instance, elevates the risk of developmental and health challenges (e.g., anxiety, hypervigilance, externalizing and internalizing behavior problems, and physiologic and neuroendocrine dysregulation) [35, 37, 38]. Parental chronic disease may also mean that offsprings assume caregiving responsibilities [39], which may hinder their ability to succeed in school, maintain peer relationships, and their own health – all of which are crucial for subsequent health trajectories [39].

There clearly is an increasing number of studies on the link between early life experiences and later life health. Yet, in resource constrained low- and middle-income countries (LMICs) like India, far less is known about the association between adverse early life factors and later life experiences of pain [40, 41]. We fill this gap in the literature by examining *(1) the prevalence of pain in older adults in India and (2) the association between early life factors and the experience of pain among older Indians*. The findings on the significance of early life exposures for later life health are particularly important for drawing conclusions about the future health of older adults in LMICs, where older adults are faced with different physical, social, and economic environments than their contemporaries in high-income nations like the US. Projections indicate an increasing aging population in LMICs, adding to the burden of chronic disease, including the experience of chronic body pain [40, 41]. As such, identifying older adults who are most susceptible to pain carries important implications for both research and policy related to later life health.

### Data and sample

The present study is based on the cross-sectional data from the baseline wave of the Longitudinal Ageing Study in India (LASI) survey, 2017–18, a nationally representative large-scale sample survey. LASI provides vital information on demography, chronic health conditions, symptom-based health conditions, functional health, mental health (cognition and depression), household economic status, healthcare utilization and health insurance, family and social networks, work and employment, retirement and life expectations of 72,250 adults aged 45 and above, across all the states and union territories of India. LASI interviewed 73,396 adults aged 45 and above (including their spouses irrespective of age) across all states and union territories of India covering 43,584 households. The detailed methodology, with the complete information on the survey design and data collection, was published in the survey report. The current study is conducted using a sample of eligible respondents, which includes older adults aged 60 years and above (n = 31,464). After removing 3,114 cases with missing information in any of the selected variables, the total sample size comprised of 28,050 older adults (men-13,509 and women-14,541).

### Outcome variable

The questions used to assess the prevalence of pain include “Are you often troubled with pain?” and “Does the pain make it difficult for you to do your usual activities such as household chores or work?” Pain was categorized as “yes” if respondents were often troubled with pain and that pain made it difficult to do usual activities such as household chores or work and “no” otherwise.

### Exposure variables

The main set of related variables consists of six different early life factors using respondents’ retrospective account of their experiences. The data on these early life factors were collected from the older respondents during the LASI survey and as such, may be subject to recall bias. The early life factors include birth order of the respondents (1 or 2, 3 or 4 and 5 and above), their childhood (before the age of 16 years) health status (good, fair, and poor), whether there were bedridden during childhood for a month or more because of health problem (yes and no), school absenteeism for a month or more because of health problem (yes and no), the respondents’ childhood financial condition (quite well-off, average, and poor), parental health problems (whether the mother or father was ever diagnosed with chronic diseases including hypertension, diabetes, heart disease, stroke, cancer, Alzheimer’s disease, Parkinson’s, and psychotic disorder). We expect these related variables to significantly determine the experience of pain in later life [17].

Additionally, we have used several conceptually relevant demographic, socioeconomic, and health variables found in the literature [23]. Age is assessed in categories (60–69 years, 70–79 years and 80 years and above), gender (male and female), education (no formal schooling, up to 5 years, 6–10 years, and more than 10 years), social group (scheduled tribes (ST), scheduled castes (SC), other backward class (OBC) and others), religion (Hindu, Muslim, and others), marital status (in a union, widowed, not in a union), place of residence (rural and urban) and wealth index assessed by monthly per capita household consumption expenditure (poor, middle, richer). Sets of 11 and 29 questions on the expenditures on food and non-food items, respectively, were used to draw the sample households. Food expenditure was collected based on a reference period of seven days, and non-food expenditure was collected based on reference periods of 30 days and 365 days. These expenditures were further standardized to a 30-day reference period and per capita monthly expenditure was computed. Our study also accounted for self-reported health (SRH) (good and poor), difficulty in activities of daily living (ADL) (yes and no), difficulty in instrumental activities of daily living (IADL) (yes and no), morbidity (none, one disease, and multiple diseases), body mass index (BMI) (underweight, normal weight, overweight, and obese), smoking (yes and no) and alcohol drinking (yes and no).

Difficulty in ADLs included asking respondents whether they had any difficulty with the following six activities: (a) walking across a room, (b) dressing, (c) bathing, (d) eating, (e) getting in and out of bed, and (f) toileting. Similarly, difficulty in IADLs was assessed by asking respondents to indicate the difficulty they encounter when performing the following seven activities: grocery shopping, preparing meals, making phone calls, taking medication, doing household chores, managing finances, and getting onself to otherwise unfamiliar location. Both ADLs and IADLs were classified as “yes” if the difficulty was reported among at least one of the respective disability forms and “no” when respondents reported having no trouble with any of the ADLs and IADLs [28]. Current morbidity was measured based on chronic ailments diagnosed, including hypertension, diabetes, cancer, chronic lung disease, chronic heart disease, stroke, arthritis, neurological/psychiatric problems, high cholesterol, thyroid, gastrointestinal problems, skin disease, and any other diseases [29]. For BMI categorization, the height and weight variables were used and measured using the standard formula.

### Statistical analysis

We used descriptive and bivariate techniques to estimate the prevalence of pain by sociodemographic variables and domains of early childhood experiences. Individual weights provided by LASI were employed to account for the multistage probability cluster sampling design and to provide the population estimates. Considering the binary nature of the outcome variable (self-reported pain), the logistic regression technique was applied to present the unadjusted and adjusted average marginal effects (AME) of selected early life factors on the probability of experiencing pain.

Marginal effect is a measurement of parameter in the probability scale, which is often a scale of interest while presenting the regression estimates. AMEs are estimated as the average difference in probability of the given outcome across all observations with covariates at their observed values. Thus, the estimates can be interpreted as the differences in the probability of the outcome in percentage points. Unlike the odds from the logistic regression, the AMEs are comparable across models, intuitively interpretable, and provide absolute measures of inequality [42]. The AME simply is the mean of all individual derivatives and can be given as:

$$ \frac{1}{n}\sum _{i=1}^{n}{\beta x}_{1}f\left({\beta x}_{i}\right)$$

where $$ {\beta x}_{1}$$ is the estimated log odds ratio for variable $$ {x}_{1}$$, $$ {\beta x}_{i}$$ is the value of the logit (i.e. the linear combination of values on variables *x* and their estimated coefficients $$ \beta $$) for the i-th observation, and $$ f\left({\beta x}_{i}\right)$$ is the probability distribution function (PDF) of the logistic distribution for $$ {\beta x}_{i}$$. In other words, ΑΜΕ expresses the average effect of $$ {x}_{1}$$ on P(*y* = 1) by taking the logistic PDF at each observation’s estimated logit, multiplying this by the coefficient for $$ {x}_{1}$$ and averaging this product across all observations [42].

In this study, AMEs from binary logistic regression should be interpreted as the difference in the probability of experiencing pain than experiences of pain in the reference group. Model 1 present the unadjusted model and model 2 is adjusted for all the covariates (exposure variables) included in this study. The results were presented in the form of AME with a 95% confidence interval (CI). The estimates are considered statistically significant at p < 0.1. All the statistical analyses were conducted using STATA (version 14) and the MS Excel program.

## Results

The characteristics of the study population are given in Table 1. 22.8% of men and 32.3% of women were troubled with pain that led to difficulty carrying out usual activities. 12.1% of the men and 14.6% of the women had mothers who suffered from chronic disease. Nearly 13% of the men and women had fathers who had a chronic disease. A proportion of 6.4% of the men and 5.1% of the women were bedridden during their childhood. Nearly 44.1% of the men and 41.7% of the women reported poor financial condition in early life.

**Table 1:** Sample and percentage distribution (column percentage) of older adults by selected background variables, stratified by sex

Background variables	Male		Female	
	Sample	Percent	Sample	Percent
**Pain**				
No	10,586	77.2	9,966	67.7
Yes	2,923	22.8	4,575	32.3
**Birth order**				
First or second	7,877	58.2	8,015	56.4
Third or fourth	3,801	28.8	4,211	27.2
Fifth and above	1,831	13.0	2,315	16.4
**Mother ever diagnosed by chronic disease**				
No	11,708	87.9	12,500	85.4
Yes	1,801	12.1	2,041	14.6
**Father ever diagnosed by chronic disease**				
No	11,639	86.7	12,740	87.0
Yes	1,870	13.3	1,801	13.0
**Childhood health status**				
Good	12,008	87.8	12,823	87.6
Fair	1,281	10.5	1,489	10.5
Poor	216	1.6	218	1.8
**Bedridden during childhood**				
No	12,759	93.6	13,864	94.9
Yes	750	6.4	677	5.1
**Missed school**				
No	13,149	97.2	13,149	99.0
Yes	360	2.9	360	1.0
**Childhood financial condition**				
Pretty well off financially	976	7.4	1,190	9.2
Average	7,112	48.5	7,654	49.1
Poor	5,421	44.1	5,697	41.7
**Age group**				
60–69 years	8,132	59.3	9,102	60.9
70–79 years	4,050	30.3	4,033	29.1
80 + years	1,327	10.4	1,406	10.0
**Marital status**				
In union	11,126	81.2	6,826	45.2
Widowed	2,035	16.4	7,354	52.9
Not in union	348	2.5	361	1.9
**Educational level**				
Illiterate	4,860	38.7	10,124	72.5
1–5 years	3,055	22.7	2,236	13.4
6–10 years	3,837	25.9	1,652	11.1
More than ten years	1,757	12.8	529	3.1
**Social group**				
Others	3,910	27.5	4,264	27.5
OBC	5,193	45.8	5,470	45.3
SC/ST	4,406	26.7	4,807	27.3
**Religion**				
Hindu	9,879	82.5	10,644	82.8
Muslim	1,618	11.1	1,704	10.7
Others	2,012	6.4	2,193	6.5
**Wealth Index**				
Poor	5,455	42.3	6,064	44.1
Middle	2,762	21.2	3,007	20.5
Rich	5,292	36.5	5,470	35.4
**Place of residence**				
Rural	9,133	73.6	9,133	69.7
Urban	4,376	26.4	4,376	30.3
**Self-reported health**				
Good	10,784	78.4	10,959	74.8
Poor	2,725	21.6	3,582	25.3
**Activities of Daily Living**				
No	11,279	80.6	11,283	75.7
Yes	2,230	19.4	3,258	24.3
**Instrumental Activities of Daily Living**				
No	8,972	62.7	7,060	44.0
Yes	4,537	37.3	7,481	56.0
**Morbidity**				
None	5,251	38.9	5,114	36.0
One disease	4,082	31.3	4,446	30.5
Multiple diseases	4,176	29.8	4,981	33.5
**Depression**				
No	12,673	92.6	13,433	90.6
Yes	836	7.4	1,108	9.5
**Body mass index**				
Underweight	3,226	28.3	3,298	25.3
Normal weight	7,590	54.2	7,118	48.5
Overweight	2,249	14.8	2,936	18.3
Obese	444	2.8	1,189	7.9
**Use tobacco**				
No	7,392	49.5	11,657	80.4
Yes	6,117	50.6	2,884	19.6
**Alcohol**				
No	11,349	86.2	14,176	98.3
Yes	2,160	13.8	365	1.7

Percentages are weighted to account for the population estimates and counts are un-weighted

**Fig. 1:**
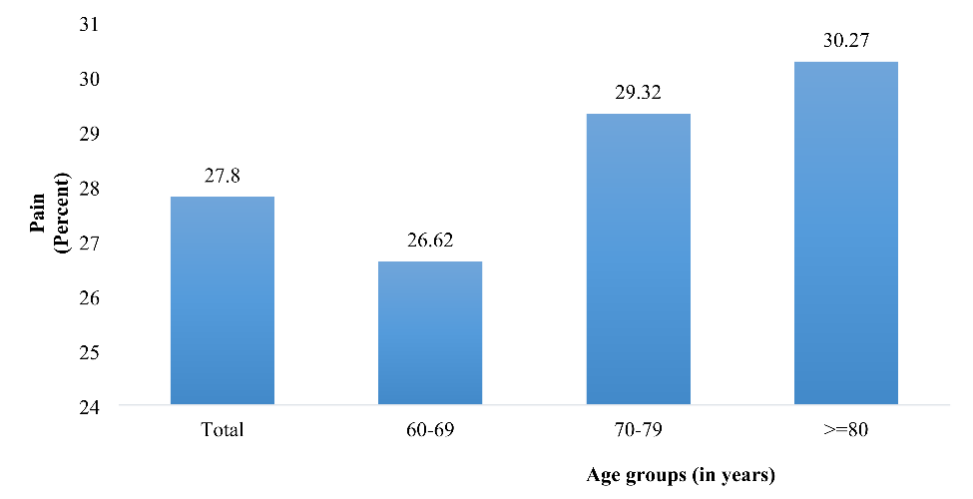
Percentage distribution of older adults experiencing pain, stratified by age groups

**Fig. 2:**
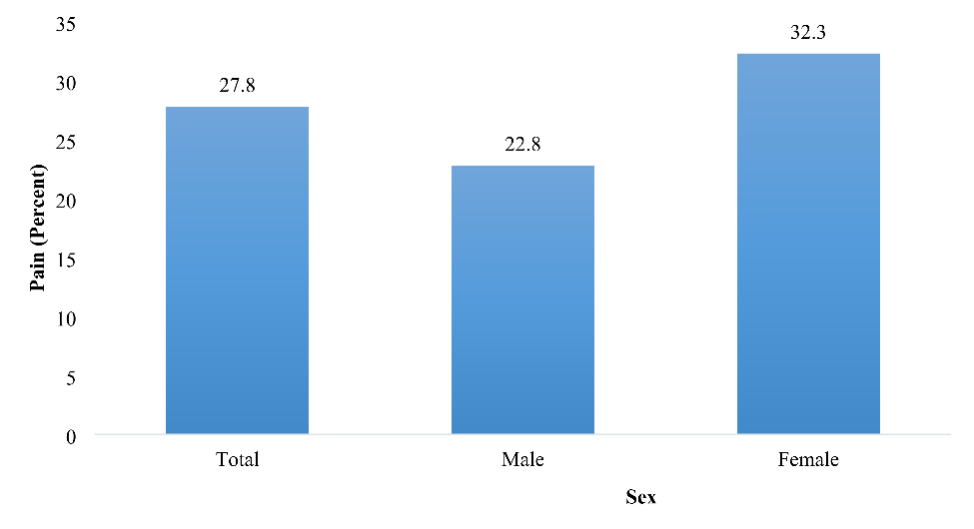
Percentage distribution of older adults experiencing pain, stratified by sex

The prevalence estimates of pain by age and gender are given in Figs. 1 and 2. Out of the study sample, 27.8% of older adults were troubled with pain. The prevalence of pain increases with age; it was 26.62%, 29.32%, and 30.27% among older adults aged 60–69 years, 70–79 years, and 80 and above, respectively. Overall, pain was higher among women (22.8%) than men (32.3%).

The prevalence of pain among older people with different characteristics is presented in Table 2. The prevalence of pain among older women, especially those who were third or fourth in birth order was higher (men = 24.3% and women = 34.5%) than among the male respondents. Older women whose mother and father suffered from chronic disease had a higher prevalence of pain than those with parents without a chronic condition. The prevalence of pain was higher among females who were bedridden during childhood as well as missed school due to sickness. The prevalence of pain was higher among women who endured financial hardship during childhood.

**Table 2:** Sample and percentage distribution (row percentage) of older adults who experience pain by selected background characteristics, stratified by sex

Background variables	Male				Female		
	Sample		Percentage	P-value	Sample	Percentage	P-value
**Birth order**				0.062			0.018
First or second	1,651		22.1		2,448	31.1	
Third or fourth	868		24.3		1,391	34.5	
Fifth and above	404		22.7		736	32.9	
**Mother ever diagnosed by chronic disease**				0.106			0.002
No	2,507		22.5		3,872	32.3	
Yes	416		24.8		703	32.1	
**Father ever diagnosed by chronic disease**				0.012			0.153
No	2,477		22.1		2,477	32.3	
Yes	446		27.2		446	32.2	
**Childhood health status**				< 0.001			< 0.001
Good	2,606		22.6		4,115	33.0	
Fair	246		22.6		368	24.0	
Poor	71		32.8		88	48.4	
**Bedridden during childhood**				< 0.001			< 0.001
No	2,703		22.2		4,294	31.7	
Yes	220		31.0		281	43.7	
**Missed school**				< 0.001			0.005
No	2,815		22.3		4,513	32.1	
Yes	108		40.2		62	49.1	
**Childhood financial condition**				< 0.001			< 0.001
Pretty well off financially	194		23.5		388	31.6	
Average	1,314		19.6		2,138	27.8	
Poor	1,415		26.2		2,049	37.7	
**Marital status**				0.056			0.045
in union	2,394		23.2		2,166	34.1	
Widowed	468		21.8		2,317	31.2	
in union	61		17.3		92	20.7	
**Educational level**				< 0.001			0.047
Illiterate	1,159		23.8		3,207	33.2	
1–5 years	747		26.2		729	35.8	
6–10 years	751		20.4		496	24.7	
More than ten years	266		18.6		143	23.7	
**Social group**				0.962			< 0.001
Others	852		23.7		1,521	36.8	
OBC	1,121		21.7		1,627	29.6	
SC/ST	950		23.9		1,427	32.3	
**Religion**				0.005			< 0.001
Hindu	2,119		22.0		3,398	31.6	
Muslim	397		29.0		587	39.8	
Others	407		22.4		590	28.8	
**Wealth Index**				0.559			0.002
Poor	1,194		22.5		1,194	31.6	
Middle	577		24.0		577	33.0	
Rich	1,152		22.5		1,152	32.7	
**Place of residence**				< 0.001			< 0.001
Rural			23.9		3,148	34.1	
Urban	789		19.7		1,427	28.3	
**Self-reported health**				< 0.001			< 0.001
Good	1,854		18.5		2,810	25.7	
Poor	1,069		38.5		1,765	51.9	
**Activities of Daily Living**				< 0.001			< 0.001
No	2,041		19.1		2,041	27.2	
Yes	882		38.2		882	48.2	
**Instrumental Activities of Daily Living**							
No	1,478	17.2			1,478	25.0	
Yes	1,445	32.1			1,445	38.0	
**Morbidity**				< 0.001			< 0.001
None	847	16.7			1,191	25.2	
One disease	859	22.5			1,336	31.0	
Multiple diseases	1,217	31.0			2,048	41.2	
**Depression**				< 0.001			< 0.001
No	2,597	21.5			4,033	30.5	
Yes	326	38.9			542	50.0	
**Body mass index**				0.043			< 0.001
Underweight	755	23.0			989	31.5	
Normal weight	1,610	22.4			2,152	32.6	
Overweight	468	23.6			1,006	32.7	
Obese	90	23.4			428	32.1	
**Tobacco**				< 0.001			< 0.001
No	1,434	20.4			1,434	30.4	
Yes	1,489	25.1			1,489	40.2	
**Alcohol**				0.044			0.352
No	2,491	23.4			4,452	32.3	
Yes	432	18.9			123	32.4	

p-values are based on Chi-Square test

The probability of experiencing pain among older adults aged 60 years and above is presented in Table 3. We used two models separately for males and females in the multivariate analysis. Model 1 shows the unadjusted AME of key early life factors (birth order, parental history of chronic diseases, childhood health status, bedridden during childhood, missed school due to illness, and childhood financial condition) on experiencing pain. Model 2 shows the adjusted AMEs of various socio-demographic characteristics on experiencing pain. The result from model 2 shows that the probability of experiencing pain was estimated to be higher for men (AME: 0.01, CI: 0.01–0.03) and women (AME: 0.02, CI: 0.01–0.04) with third or fourth birth order, respectively compared to men and women with first birth order. Although not significant in the adjusted model, the probability of experiencing pain was higher among women whose mother was diagnosed with any chronic disease, at same point, it was higher among men, whose father was diagnosed with any chronic disease. Men with poor childhood health status are estimated to have a higher probability of experiencing pain, while at the same time, both men (AME: -0.02, CI: -0.04–0.01) and women (AME: -0.07, CI: -0.09 - -0.04) having fair childhood health status had a lower probability of experiencing pain. The probability of experiencing pain was estimated to be higher among both men (AME: 0.03, CI: 0.01–0.07) and women (AME: 0.07, CI: 0.03–0.13) who were bedridden due to sickness in their childhood. Similarly, the probability of experiencing pain was higher among men who missed school for more than a month due to health problem (AME: 0.04, CI: -0.01-0.09) and it was not significant in the case of women. Men and women with poor financial condition in their childhood were estimated to have higher probability of experiencing pain (AME: 0.04, CI: 0.01–0.07) compared to their peers who reported more financially advantaged early life.

**Table 3:** Average marginal effects (AME) of experiences of pain among older adults aged 60 years and above in India, stratitified by sex, LASI Wave 1, 2017-18 (N = 28,050)

Background variables	Male		Female	
	Model 1	Model 2	Model 1	Model 2
	AME (CI at 95%)	AME (CI at 95%)	AME (CI at 95%)	AME (CI at 95%)
**Birth order**				
First or second	Reference	Reference	Reference	Reference
Third or fourth	0.02**(0.01 0.03)	0.01*(0.01 0.03)	0.03***(0.01 0.04)	0.02**(0.01 0.04)
Fifth and above	0.01 (-0.01 0.03)	0.01 (-0.01 0.03)	0.01 (-0.01 0.04)	0.01 (-0.02 0.02)
**Mother ever diagnosed by chronic disease**				
No	Reference	Reference	Reference	Reference
Yes	0.01 (-0.01 0.03)	0.01 (-0.02 0.02)	0.04***(0.01 0.06)	0.01 (-0.02 0.02)
**Father ever diagnosed by chronic disease**				
No	Reference	Reference	Reference	Reference
Yes	0.03**(0.01 0.05)	0.01 (-0.01 0.03)	0.01 (-0.02 0.03)	-0.02 (-0.04 0.01)
**Childhood health status**				
Good	Reference	Reference	Reference	Reference
Fair	-0.03**(-0.05 -0.01)	-0.02**(-0.04 0.01)	-0.08***(-0.10 -0.05)	-0.07***(-0.09 -0.04)
Poor	0.07**(0.01 0.13)	0.01 (-0.04 0.06)	0.05 (-0.02 0.11)	0.01 (-0.06 0.05)
**Bedridden during childhood**				
No	Reference	Reference	Reference	Reference
Yes	0.06***(0.02 0.09)	0.03**(0.01 0.07)	0.09***(0.05 0.13)	0.07***(0.03 0.11)
**Missed school**				
No	Reference	Reference	Reference	Reference
Yes	0.03 (-0.02 0.08)	0.04*(-0.01 0.09)	0.04 (-0.04 0.12)	0.02 (-0.06 0.09)
**Childhood financial condition**				
Pretty well off financially	Reference	Reference	Reference	Reference
Average	-0.01 (-0.04 0.02)	0.01 (-0.03 0.02)	-0.04***(-0.07 -0.01)	-0.02 (-0.05 0.01)
Poor	0.07***(0.04 0.09)	0.04***(0.01 0.07)	0.04***(0.01 0.07)	0.04***(0.01 0.07)
**Age group**				
60–69 years		Reference		Reference
70–79 years		0.01 (-0.01 0.02)		0.01 (-0.01 0.03)
80 + years		0.01 (-0.02 0.02)		-0.02*(-0.05 0.01)
**Marital status**				
In union		Reference		Reference
Widowed		-0.01 (-0.03 0.01)		-0.03***(-0.05 -0.02)
Not in union		-0.05**(-0.09 -0.01)		-0.07***(-0.11 -0.02)
**Educational level**				
Illiterate		Reference		Reference
1–5 years		0.01 (-0.01 0.03)		0.01 (-0.02 0.03)
6–10 years		-0.02*(-0.03 0.01)		0.01 (-0.02 0.03)
More than ten years		-0.04***(-0.06 -0.01)		-0.01 (-0.05 0.03)
**Social group**				
Others		Reference		Reference
OBC		-0.02*(-0.03 0.01)		-0.06***(-0.08 -0.05)
SC/ST		-0.01 (-0.02 0.01)		-0.04***(-0.06 -0.02)
**Religion**				
Hindu		Reference		Reference
Muslim		0.02*(0.01 0.04)		0.01 (-0.02 0.02)
Others		0.02 (0.01 0.04)		-0.03***(-0.05 -0.01)
**Wealth Index**				
Poor		Reference		Reference
Middle		0.01 (-0.02 0.02)		0.01 (-0.02 0.01)
Rich		0.01 (-0.01 0.02)		0.01 (-0.01 0.02)
**Place of residence**				
Rural		Reference		Reference
Urban		-0.04***(-0.05 -0.02)		-0.06***(-0.07 -0.04)
**Self-reported health**				
Good		Reference		Reference
Poor		0.13***(0.11 0.15)		0.16***(0.14 0.18)
**Activities of Daily Living**				
No		Reference		Reference
Yes		0.10***(0.08 0.13)		0.10***(0.08 0.12)
**Instrumental Activities of Daily Living**				
No		Reference		Reference
Yes		0.06***(0.05 0.08)		0.06***(0.04 0.07)
**Morbidity**				
None		Reference		Reference
One disease		0.03***(0.01 0.05)		0.05***(0.03 0.06)
Multiple diseases		0.08***(0.06 0.10)		0.12***(0.10 0.14)
**Depression**				
No		Reference		Reference
Yes		0.08***(0.05 0.11)		0.10***(0.07 0.12)
**Body mass index**				
Underweight		Reference		Reference
Normal weight		0.01 (-0.01 0.02)		0.02*(0.00 0.03)
Overweight		0.01 (-0.01 0.03)		0.05***(0.02 0.07)
Obese		0.01 (-0.03 0.05)		0.05***(0.02 0.08)
**Tobacco**				
No		Reference		Reference
Yes		0.04***(0.03 0.06)		0.05***(0.03 0.07)
**Alcohol**				
No		Reference		Reference
Yes		-0.02**(-0.04 0.00)		0.03 (-0.02 0.08)

Note: AME denotes averaged marginal effects that represent the probability of experiencing pain obtained from logistic regressions, * if p < 0.1, ** if p < 0.05, *** if p < 0.01. Model 1 is unadjusted model and model 2 is adjusted for all the covariates included in Table 2

## Discussion

Both stress process and life course scholars have underscored that adversity in early life is negatively consequential for health in later life [35, 38, 43]. That said, relatively less attention is given to pain experienced by older adults, and especially so in LMICs like India [40, 41]. This is problematic given that as the world’s aging population grows, so will the prevalence of pain [44, 45]. The present study fills this gap in the literature by studying the prevalence of pain in older adults in India. Crucially, we also investigate the association between a number of early life factors and older Indian’s experiences with pain.

Our findings show that unfavorable early life circumstances are strongly and negatively correlated with pain among older persons in India, even after adjusting for a number of conceptually relevant socioeconomic, demographic, and health factors. Several processes may link early life situations to pain in later life. According to studies based on the critical model [46, 47], biological changes brought on by early-life experiences may have a long-term impact on one’s health and may even be irreversible. Alternatively, proponents of the pathway model contend that early life circumstances influence both hazardous and beneficial exposures in later life, which in turn affect older people’s health and well-being [46, 48].

We find that older adults with lower early life SES reported a higher prevalence of pain than their peers with more financially advantaged beginnings. While those living in higher SES households may have access to better education, health care, and good quality jobs and housing, their lower SES peers may lack such benefits and instead may get exposed to greater levels of conflict, food and housing insecurity, substance use, and other forms of socioeconomic disadvantage [49]. Such disparate early life experiences accrued due to lower SES may only widen disparities in health conditions, including pain, later in life. Another mechanism linking early life SES and later life pain is health behaviors. Higher childhood SES is associated with health-promoting behaviors (e.g., reduced smoking, moderate drinking, regular exercise, and balanced nutrition), whereas lower childhood SES is linked to behaviors that may be deleterious to later life health [48, 50, 51].

In addition to lower SES, our study also revealed a statistically significant association between childhood health problems and the experience of pain in later life. Whereas older men and women with fair childhood health reported a lower likelihood of pain, older males with poor early life health reported a higher incidence of pain. Moreover, both men and women who were bedridden as children due to sickness had a higher prevalence of pain. These findings are comparable to those found in previous studies [52, 53]. Children who are exposed to illnesses may experience detrimental effects on their physical, mental, and cognitive development, restricting their opportunities to secure a quality education [54]. Low education is typically associated with a lower SES, which can restrict access to resources and health care services, leading to poor health. Treatment compliance may also be hampered by limited education, leading to impaired communication with medical personnel [55]. Lastly, childhood ill health is linked to an increased risk of mental distress, namely depressive symptoms [56]; depression, in turn, is associated with increased sensitivity to pain [57].

As expected, we also discovered a link between parental chronic illness and the prevalence of pain in later life. The prevalence of pain was higher among older women whose mothers suffered from chronic condition, while pain prevalence was higher among older men who reported having had fathers with chronic illness. Chronic illness may disrupt parents’ ability to cope with daily stressors and contribute to hostility, harsh parenting, disengaged care, and compromised parent-child interactions, which in turn can lead to feelings of uncertainty, insecurity, and mental distress [58]. Teenagers with chronically ill parents are significantly more likely than other children to internalize their issues (i.e., self-blame, rumination, hopelessness, socially withdrawn behavior, and somatic complaints) [59–61], which may have long-term health effects, including the experience of pain in later life. The link between parental disease and pain in later life may also be caused by a shared genetic component, an acquired pain behavior, or environmental circumstances that promote pain in both parents and children [62]. These elements may influence how symptoms are interpreted, the propensity towards certain coping mechanisms, and pain vulnerability [62].

Our study, like previous ones [4, 63], found older women more likely to report pain than their male peers. Additionally, the associations between higher birth order, financial hardship, bedriddenness, and school absenteeism due to illness, exposure to parental chronic illness, and the likelihood of experiencing pain in later life were considerably more pronounced among older women than men. While the precise mechanisms causing gender disparities in pain are unknown, it has been postulated that biological, psychological, and sociocultural elements interact to cause these discrepancies [64]. Differences in sex hormone levels, how the brain interprets pain-related signals, and the perceptions and interpretations related to pain may all contribute to gender disparities in pain [65–67]. For instance, women frequently experience pain more intensely [66, 68] than men and typically show less activation in brain regions that regulate pain [69]. Also, how individuals express pain may be influenced by the cultural ideals of masculinity and femininity [63, 68]. Such ideals also may permit women, but not men, to report pain, contributing to the observed discrepancies.

The findings of our study should be interpreted within the context of some important limitations. *First*, the cross-sectional nature of LASI precludes us from drawing any causal or temporal inferences about the primary variables of interest. Future investigations should rely on longitudinal data with repeated measures and time-varying covariates for assessing the linkages between early life factors and related demographic, social, and behavioral factors contributing to chronic pain over time. *Second*, LASI precludes us from gauging either the type of pain (e.g., back, neck, full body pain, etc.) or severity of it. Further, information on age at onset of pain or the perceived tolerance associated with pain also is unavailable. Future studies replicating our work should consider a more comprehensive assessment of the experience of pain as it relates to early life factors. *Third*, our findings rely on several self-reported measures of health, including subjective assessment of pain, which may inflate some of the association between early life factors and subsequent experience of pain. That said, the reliability of self-rated health in predicting a myriad physical and mental health problems and even mortality is unusually strong and reported widely in numerous epidemiological and medical studies [70–72]. *Fourth and related*, early life experiences also are measured using retrospective self-recall. Despite the findings concerning potential recall bias, studies have revealed a trend of significant under-reporting rather than over-reporting of childhood adversity [73, 74]. *Fifth*, despite the exhaustive range of covariates in our study, the problem of residual confounding due to unmeasured variables remains. Future studies should consider the timing of onset and duration of adversity as they may be critical in determining the accumulation of social and psychological resources needed to maintain good health. For example, a more complete medical history of parents’ health would be necessary to explore whether individuals are sensitive to the timing of exposure to this stressor [38]. Further, while present research assesses socio-environmental correlates of later life pain, chronic pain like other chronic health conditions may also be linked to genetic pre-dispositions [75]. Future scholarship, we recommend, should consider such factors to help design more appropriate interventions for the aging population.

Notwithstanding these caveats, the current study serves as a starting point for future research on the link between early life events and chronic pain in later life. While the majority of studies in this area are based on samples of older adults in high-income western nations, we focus on the association between early life factors and later life pain among older adults in India. In fact, our work is among the first of its kind within the Indian context to assess the implications for later life experience of pain of early life conditions. We do so by employing a large and nationally representative sample of older Indians.

## Conclusion

In sum, our study finds early-life influences to be consequentially linked to the experience of pain among older Indians. Our findings are consistent with the life course perspective, which highlights that stress exposure during critical periods, such as childhood and adolescence, can cascade into unfavorable health outcomes for individuals throughout their lives, including later life [76, 77]. Public policy on aging, as such, should make investments in individuals at earlier periods of their life as early life experiences are associated with subsequent experiences of pain.

## Data Availability

The data are available at The Gateway to Global Aging Data (https://g2aging.org/ ).
